# Disease Management Maintains Adequate Chlorophyll *a* Fluorescence and Enhances Wheat Grain Technological Quality

**DOI:** 10.3390/plants15050688

**Published:** 2026-02-25

**Authors:** Andrea Román, Carlos Eduardo Aucique-Perez, Martha Zavariz de Miranda, Pihetra Oliveira Tatsch, Eduardo Rodríguez, Leandro José Dallagnol

**Affiliations:** 1 Laboratory of Plant Pathogen Interaction, Crop Protection Department, Eliseu Maciel Faculty of Agronomy, Federal University of Pelotas, Pelotas 96010-900, Rio Grande do Sul, Brazil; aroman@ueb.edu.ec; 2 Agricultural Sciences Natural Resources and the Environment Faculty, Bolivar State University, Guaranda EC020150, Ecuador; eduguez83@gmail.com; 3 Horticultural Science Department, North Florida Research and Education Center, University of Florida/IFAS, Quincy, FL 32351, USA; c.auciqueperez@ufl.edu; 4 Brazilian Agricultural Research Corporation, Embrapa Trigo, P.O. Box 78, Passo Fundo 99050-970, Rio Grande do Sul, Brazil; martha.miranda@embrapa.br (M.Z.d.M.); pihetra.tatsch@embrapa.br (P.O.T.); 5 Grow Green Agricultural Technologies, Riobamba EC060103, Ecuador

**Keywords:** *Triticum aestivum*, dough rheology, ash, nitrogen, leaf diseases, F_v_/F_m_

## Abstract

Leaf and spike diseases can significantly reduce wheat yield and grain quality. To mitigate these impacts, an integrated disease management approach can be adopted, incorporating measures such as the use of resistant cultivars, fungicides and nitrogen fertilization. This study aimed to evaluate the impact of these practices on chlorophyll *a* fluorescence, yield components, and the technological quality of wheat grains. The area under the disease progress curve (AUDPC) was correlated with the maximum efficiency of photosystem II (PSII) photochemistry (F_v_/F_m_), as measured at the dough development stage (ZGS80) under field conditions, which also affected quality parameters. Additionally, an increase in AUDPC values reduced the thousand kernel weight (TKW) and test weight (TW). Conversely, AUDPC values for tan spot, powdery mildew and leaf rust were positively related to ash content (affecting flour color), protein content (PC) and grain falling number. Both the recommended nitrogen rate (130 kg ha^−1^) and the high rate (200 kg ha^−1^) increased grain protein content (PC) and gluten index (GI), while maintaining dough stability and water absorption. Fungicide application increased flour lightness and yellowness. Overall, integrated disease management combining moderately resistant cultivars, fungicide applications and nitrogen fertilization reduced AUDPC values, increased F_v_/F_m_ (indicating optimal physiological performance) and ensured yield components and maintenance of wheat technological quality.

## 1. Introduction

Wheat *(Triticum aestivum* L.) was the most consumed cereal worldwide [1], with an estimated demand of 798.3 million tons by 2024 [2]. In Brazil, it was estimated that production would reach 8.1 million tons in 2024, whereas national demand would reach 11.9 million tons, representing a 31.9% production deficit [3,4]. In this context, increasing wheat production through more efficient and sustainable disease management tools is essential [5] to ensure both yield and grain quality.

Several biotic factors caused by pathogens during plant development threaten wheat grain yield and technological quality. These include leaf diseases caused by *Pyrenophora tritici-repentis* (Died.) Drechs (tan spot), *Puccinia striiformis* (stripe rust), *Puccinia triticina* (leaf rust), *Blumeria graminis* (DC.) f. sp. *tritici* (powdery mildew), as well as spike diseases such as those caused by the *Fusarium graminearum* complex (Fusarium head blight—FHB) and *Magnaporthe oryzae* pathotype *Triticum* (blast). Leaf spot, powdery mildew, and rust reduce the photosynthetic leaf area, accelerate leaf senescence, and ultimately lower grain yield [6]. Yield losses of up to 75% due to tan spots, 3 to 25% due to FHB, 40% from powdery mildew, and up to 90% from rusts have been recorded [7,8,9,10].

Powdery mildew, for example, reduces leaf assimilation capacity and compromises grain yield by limiting photosynthetic efficiency [11,12]. Pathogen infection of leaves and spikes may result in shriveled grains, thereby lowering the flour extraction efficiency in milling [13]. Infection by *P. striiformis* also impairs photosynthetic function and accelerates the translocation of assimilates from leaves to grain, resulting in lower thousand kernel weight (TKW) and reduced yield [14].

Grains from wheat plants affected by diseases such as tan spot, rust, and FHB generally present lower protein and gluten contents, along with impaired rheological parameters and viscoelastic properties [15,16,17]. Some studies have shown that changes in viscoelastic properties result from altered gluten protein polymerization in dough, caused by biotic stress, especially from diseases [18,19,20]. Furthermore, FHB poses a major threat to cereal production, as it not only reduces yield but also contaminates grains with mycotoxins, compromising food safety and posing risks to both human and animal health [21].

To mitigate yield and quality losses, integrated disease management practices have been widely adopted, including the use of resistant genotypes, healthy seeds, fungicides and fertilizers [16,22,23,24]. These strategies not only aid in disease control but also influence the wheat technological quality [25]. Agronomic traits, such as flowering time and plant stature are also essential for crop adaptation and yield potential [26]. Early-maturing cultivars, in particular, are advantageous as they enable multiple cropping per season and reduce the risks associated with environmental stresses during grain filling and maturation [27].

Fungicide pre-mixes with different modes of action (azoles, strobilurins, and carboxamides) have significantly improved grain yield and grain quality parameters, including thousand kernel weight (TKW), test weight (TW), and grain starch content (GSC) [28]. When combined with higher nitrogen (N) rates, these pre-mixes also reduce damage caused by Septoria tritici blotch (*Zymoseptoria tritici*) and tan spot while further increasing the yield [15,16,29].

Chlorophyll *a* (Chl*a*) fluorescence is used as indicator of plant physiological status. Specifically, the maximum efficiency of photosystem II (PSII) photochemistry (F_v_/F_m_*)*, also known as the maximum quantum yield of primary photochemistry [30,31,32], serves as a marker of photoinhibition and stress levels [31,33,34]. In non-stressed plants, F_v_/F_m_ values typically range from 0.80 to 0.83, whereas stressed plants exhibit pronounced reductions due to photoinhibition and PSII impairment [35]. Several studies have reported reduction in F_v_/F_m_ in response to biotic stress across multiple pathosystems [36,37]. Thus, this parameter enables linking disease-induced damage to photosynthetic performance, particularly at the PSII level, as well as to wheat technological quality parameters assessed through physicochemical and rheological analyses.

Although several studies have investigated the effects of N and fungicide applications on disease management and technological quality [15,16,38,39,40], information regarding changes in PSII and wheat technological quality parameters in response to disease management in early-maturing wheat cultivars under field conditions remains limited. Therefore, the objective of this study was to determine the effects of disease management measures on Chl*a* fluorescence, yield components, and wheat technological quality.

## 2. Results

### 2.1. Effect of Cultivar, Fungicide Application, and Nitrogen Rate on Wheat Physicochemical and Rheological Quality

In this experiment, we observed the effects of cultivar, nitrogen rate, and fungicide application for disease management on the technological quality during the growing season 2019. The cultivar, fungicide, and nitrogen treatments were significant (*p* < 0.05) for TKW, TW, AC, GFN, PC, IG, and F_v_/F_m_, while fungicide was significant for TKW, TW, and AC (Table 1). Nitrogen levels were significant for TKW, TW, PC, and IG (*p* < 0.05) (Table 1).

In 2019, TKW, TW, and GI were 15%, 4%, and 29.5% higher, respectively, in TBIO Audaz compared with TBIO Tibagi, whereas AC (10.7%) and PC (8%) were higher in TBIO Tibagi than for TBIO Audaz (Table 1). A similar trend was observed in 2020: TKW, TW, and GI were again higher in TBIO Audaz compared with TBIO Tibagi, while AC and PC remained higher in TBIO Tibagi (Table 2). Regarding fungicide application, AC was 9% higher under the non-sprayed condition, whereas fungicide treatment increased TKW and TW by 15% and 7%, respectively. Under higher nitrogen fertilization, increases of 5% in TKW, 13% in PC, and 11% in GI were observed.

Conversely, in 2020, TKW, TW, GFN, and F_v_/F_m_ were 25%, 5%, 1%, and 14% higher, respectively, when fungicide was applied. Under the non-sprayed condition, AC, PC, and GI increased by 4%, 7.1%, and 9%, respectively (Table 2). Regarding nitrogen, a similar trend to that observed in 2019 was recorded: TKW and PC increased by 3.5% and 6.7%, respectively, at the highest nitrogen rate. However, AC was 4% higher under the lowest nitrogen rate compared with the highest rate.

### 2.2. Effect of Cultivar, Fungicide Application, and Nitrogen Rate on Flour Color Parameters

The effect of cultivar was significant (*p* < 0.05) for all flour color parameters (L*, a*, b*, C*, and h*) (Table 3). In 2019, TBIO Tibagi showed 5% and 0.4% higher L*, and h* values, respectively, compared with TBIO Audaz. Conversely, a*, b*, and C* were 25%, 23%, and 22% higher, respectively, in TBIO Tibagi than in TBIO Audaz.

When fungicide was applied, L*, and h* increased by 1% relative to the unsprayed plants, and similar results were observed in 2020 (Table 4). In contrast, unsprayed treatments showed higher a*, b*, and C* values, with increase of 11 and 4% in 2019 (Table 3), and 11 and 6% in 2020, respectively, compared with fungicide-treated plots (Table 4).

The nitrogen rate significantly affected L*, a*, and h* values in 2019 (Table 3). For L*, and h*, low N resulted in values approximately 1% higher than those observed under the high N rate. In contrast, a* increased by 5% under the high N rate compared with the low N rate (Table 3). However, in 2020, the N rate did not significantly influence any of these color parameters (Table 4). Regarding interactions, the effects were inconsistent between years. Interactions between cultivar × fungicide and fungicide × nitrogen rates varied across parameters, indicating that the individual factors exerted stronger and more consistent effects than their combinations (Table 4). Overall, TBIO Tibagi produced flours with high L*, and reduced redness (a*) and yellowness (b*) compared with TBIO Audaz. Fungicide application increased flour color saturation and intensity, reflected in higher hue and chroma, respectively, resulting in enhanced lightness and a stronger yellow tendency. Similarly, a low N rate increased both L* and h* (Table 4).

### 2.3. Correlation Analysis: AUDPCs, Chla Fluorescence, Yield Components, and Wheat Grain Technological Quality

**Figure 1:**
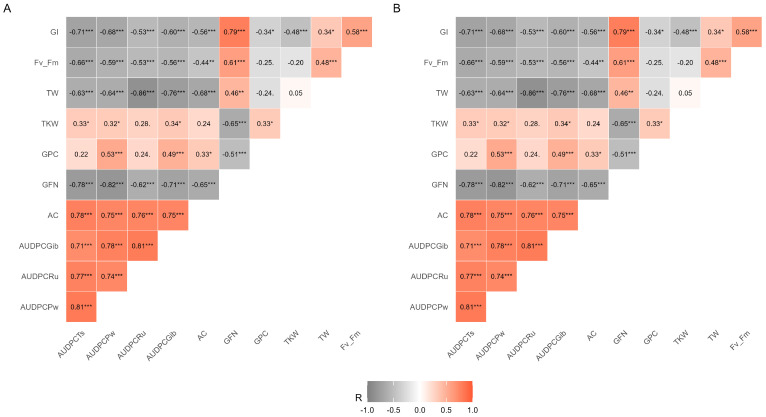
Correlation matrix among the attributes evaluated in this study during 2019 (**A**) and 2020 (**B**). Area under disease progress curve of tan spot (AUDPCTs), powdery mildew (AUDPCPw), FHB (AUDPCGib), rust (AUDPCRu), ash content (AC), grain falling number (GFN), grain protein content (PC), gluten index (IG), thousand kernel weight (TKW), test weight (TW), and maximum efficiency of PSII photochemistry (F_v_/F_m_*).* *** *p* ≤ 0.001 highly significant; ** *p* ≤ 0.01 significant; * *p* < 0.05 significant.

The correlation matrix showed that AUDPCTs were positively correlated with AC (Figure 1A,B). Both AUDPCTs and AUDPCPw were negatively correlated with TKW, TW, and yield, while AUDPCPw also showed a negative correlation with GI. AUDPCRu was positively correlated with AC and GFN (Figure 1). In 2019, AUDPCGib was negatively correlated with GFN (Figure 1A); however, this effect was not observed in 2020 (Figure 1B). Both AUDPCTs and AUDPCPw were negatively associated with F_v_/F_m_ (Figure 1A,B). AC and GFN were negatively correlated with TKW, TW, and yield, although the magnitude of those relationships varied between years (Figure 1). GI was negatively associated with PC, whereas TKW showed positive correlation with TW, and yield was positively correlated with both TKW and TW (Figure 1A,B). Regarding wheat grain technological quality, GFN and GI were positively correlated, while PC showed a negative correlation (Figure 1).

## 3. Discussion

In this study, disease occurrence was registered during the 2019 and 2020 growing seasons, and disease management practices influenced both the maximum efficiency of PSII photochemistry (F_v_/F_m_) and wheat technological quality variables. TBIO Tibagi, the susceptible cultivar, was most affected in both years. The reduction in F_v_/F_m_ was associated with the increase in AUDPCTs and AUDPCPw. In these contexts, previous studies have similarly reported that reductions in F_v_/F_m_ across different pathosystems are associated with pathogen infection [33,37,41,42]. The use of a resistant cultivar, fungicide and nitrogen can mitigate disease impacts without severe reduction in F_v_/F_m_. Particular, the fungicide pre-mix reduced disease damage and was associated with a higher F_v_/F_m_, consistent with Ajigboye et al. [43], who reported that succinate dehydrogenase inhibitor (SDHI) and triazole fungicide increased PSII efficiency and enhanced photosynthesis during grain filling, resulting in improved yields.

The negative correlation from AUDPCTs and AUDPCPw with TKW, TW, and yield highlights the detrimental effects of diseases on these parameters. In 2019, TBIO Tibagi (susceptible) showed lower TKW and TW than TBIO Audaz (moderately resistant), but fungicide application increased these parameters in TBIO Tibagi, suggesting that tan spot and powdery mildew were largely responsible for reductions. The fungicide pre-mix alleviated these losses, consistent with reports by Matzen et al. [28] and Kutcher et al. [44]. Similarly, recommended and high N rates increased TKW, and the combination of N application with fungicides reduced disease damage and improved grain yield, as reported in other studies [15,45,46].

The associations between F_v_/F_m_ and AUDPCs values further demonstrate that reduced PSII efficiency is linked to disease severity and correlated with changes in technological quality traits. AUDPC values of tan spot, powdery mildew, and leaf rust were positively associated with AC, which in turn was linked to reduced test weight, flour contamination and altered flour color. Fungicide and higher N rates decreased wheat flour AC, improving it to desirable levels (<2%), consistent with baking quality standards [47]. AUDPC values were negatively correlated with GFN, suggesting that alpha-amylase activity is influenced by the cultivar genetic background, fertilization, and crop management [28,48,49]. The delayed leaf senescence and reduced disease in TBIO Audaz were associated with a higher GFN compared with the susceptible cultivar (TBIO Tibagi), although both remained above 250 s, the threshold required for breadmaking [50].

Disease management through cultivar, nitrogen, and fungicide application reduced disease intensity, with positive effects on grain protein content (PC) and gluten parameters. The positive association of AUDPCPw with PC and its negative association with F_v_/F_m_ indicate that disease severity reduces PC. Differences between cultivars also indicated that yield gains can dilute protein concentration, reducing grain quality. TBIO Audaz exhibited higher TW and TKW, but lower PC than TBIO Tibagi, a trend consistent with the protein dilution effect reported by other authors [17,51,52].

In this regard, delay in senescence and extension of the grain-filling period of cereals due to fungicides (strobilurins and triazoles), along with genetic improvement of cultivars, may be associated with effects on grain quality [53], suggesting that the use of fungicides and appropriate N rates allow adequate disease control while maintaining protein and gluten content.

This effect was observed in both cultivars that achieved higher PC, DG and WG under high nitrogen (N) rates, whereas low N reduced these traits, particularly in the TBIO Tibagi cultivar, where low N rates limited nitrogen assimilation due to susceptibility to disease, thereby affecting protein and gluten content. These findings are consistent with previous studies that have reported reductions in protein and gluten levels due to leaf diseases [28,46]. However, the combination of higher N and a fungicide pre-mix was found to further improve protein and gluten content, confirming synergistic effects [15,38]. Moreover, proper N fertilization and fungicide application maintained protein and gluten at desirable levels while improving rheological properties such as water absorption and dough stability, which reflect dough resistance to mixing.

Rheological analysis also confirmed that fungicides application under low N availability resulted in weak flours with reduced baking potential. However, recommended or high N rates mitigated this effect by compensating for N immobilization caused by delayed senescence and prolonged green leaf area, as previously reported [38,52]. This result is further associated with higher F_v_/F_m_, resulting from low tan spot severity, as reported in previous experiments [34].

Finally, the color analyses demonstrated the complementary roles of cultivar, fungicide, and N management in determining flour quality. Disease-related reduction in F_v_/F_m_ were mirrored by decreases in L*, a*, b*, and C*. Increases in protein, gluten, and AC were associated with reductions in b* and C*, whereas variation in GFN reduced L*. Adequate N supply under fungicide application improved dough strength, stability, and farinograph quality number, whereas low N combined with no fungicide produced weaker flours with reduced baking potential. Similarly, fungicide application and higher N improved flour color parameters (L*, a*, b*, C*, and h*) enhancing visual quality. Overall, these results demonstrate that integrated disease management, through cultivar resistance, proper fungicide use, and adequate N supply, simultaneously improves physiological performance, yield components, and the technological and visual quality of wheat flour.

## 4. Materials and Methods

### 4.1. Experimental Materials

Two early-maturing wheat (*Triticum aestivum*) cultivars, TBIO Audaz (Biotrigo^®^, Passo Fundo, Brazil) and TBIO Tibagi (Biotrigo^®^), were selected among regionally adapted cultivars with similar anthesis and maturity dates. The cultivars also differ in disease resistance: TBIO Audaz is more resistant to tan spot, powdery mildew, leaf rust, and FHB than TBIO Tibagi, according to Román et al. [34].

### 4.2. Agronomic Field Trails

Field experiments were conducted at the Palma Agricultural Center (31°48′06.4″ S 52°30′18.6″ W), of the Federal University of Pelotas, Brazil, during two growing seasons (2019 and 2020). Plots were established in an area previously under a wheat–soybean succession in 2018. Sowing was performed under no-till conditions on 11 July 2019 and 14 July 2020 using a plot seeder (Semeato, SHP model, Passo Fundo, Brazil) at a density of 300 seeds m^−2^ to achieve approximately 275 plants m^−2^. Each plot consisted of nine rows spaced 0.17 m apart. Soil characteristics are presented in Table S1. Weather data (daily rainfall; relative humidity; minimum, maximum, and mean temperatures) were obtained from the Pelotas weather station, located at latitude 31°52′00″ S and longitude 52°21′24″ W and an altitude of 13.24 m (Table S2).

### 4.3. Experimental Design

The experiment followed a randomized block design with three factors and four replicates. The factors included (1) fungicide treatment (sprayed, S; or non-sprayed, NFS), (2) wheat cultivars, and (3) nitrogen levels: low (LN, 70 kg ha^−1^), recommended (RN, 130 kg ha^−1^), and high (HN, 200 kg ha^−1^).

### 4.4. Agronomic Management

Diseases developed naturally from field inoculum. Base fertilization followed the soil-test recommendation: phosphorus at a rate of 40 kg ha^−1^ in the form of triple superphosphate and potassium at a rate of 30 kg ha^−1^ in the form of potassium chloride at sowing. Weed management included glyphosate [Shadow 480 SL^®^ herbicide (Albaugh AgroBrasil Ltd.a, São Paulo, Brazil), 480 g L^−1^, 2 L ha^−1^] applied 15 days before sowing, and metsulfuron-methyl [Zartan^®^ herbicide (UPL), Ituverava, Brazil; 600 g kg^−1^, 0.006 g ha^−1^] at the three-leaf stage (ZGS13).

Fungicide applications consisted of a pre-mix of bixafen (125 g L^−1^; carboxamide) + prothioconazole (175 g L^−1^; triazolinthione) + trifloxystrobin (150 g L^−1^; strobirulin) (Fox Xpro^®^; Bayer, São Paulo, Brazil) at 0.5 L ha^−1^ applied at flag leaf emergence (ZGS39) and at flowering (ZGS65) [Zadoks stages]. Applications were performed using a CO_2_ pressure sprayer equipped with four nozzles (TTJ60 11002; Teejet^®^, North Sioux City, SD, USA) delivering 200 L ha^−1^. Nitrogen was applied as urea (45%) split between sowing and tillering (ZGS23). Each sub-subplot was 3 m^2^ (2 m long × 1.5 m wide).

### 4.5. Experimental Measurements

#### 4.5.1. Disease Assessment and Area Under Disease Progress Curve (AUDPC)

Disease severity was assessed for tan spot, powdery mildew, leaf rust, and FHB. Ten central plants per plot were evaluated for foliar diseases (four leaves plus the flag leaf), and 20 spikes were assessed for Fusarium head blight (FHB). Diseases monitoring was performed from stem elongation (ZGS30) to the hard dough stage (ZGS87). Tan spot and powdery mildew severities were rated using the Horsfall–Barratt scale [54], leaf rust using the modified Cobb [55], and FHB using the Stack and McMullen scale [56]. For each disease, the AUDPC was calculated following Shaner and Finney [57], and later correlated with technological quality traits.

#### 4.5.2. Determination of Chlorophyll (Chl) *a* Fluorescence (CF)

CF was measured in leaves previously dark-adapted (30 min) using a handheld fluorometer (FluorPen FP110; Photon Systems Instruments, Drásov, Czech Republic) from 5:30 to 6:30 a.m. Polyphasic fluorescence transients (OJIP) were induced by saturating light at 3000 µmol m^−2^ s^−1^, and the fluorescence kinetics were recorded. Measurements were performed according to Ajigboye et al. [33], including fluorescence at 50 ms, considering the initial fluorescence value (F_0_). The maximum fluorescence level of the OJIP transient (F_m_) was measured under saturating light conditions. Intermediate fluorescence values were measured at 300 ms, 2 ms, and 60 ms and labeled as F_300ms_, F_j_ and F_i_, respectively, as shown by Strasser et al. [30]. The different steps of the OJIP transient were determined using FluorPen 1.0.0.6 software (Photon Systems Instruments, Drásov, Czech Republic). The ZGS80 observation was chosen after previous analyses (Figures S1 and S2). Only the maximum quantum yield of the primary photochemistry (F_v_/F_m_) was used for the analysis.

#### 4.5.3. Yield Determinations

Wheat was harvested from five central rows with a length of 1 m (totaling 1 m^2^) in each plot and threshed with a mechanical grain thresher (EDA, model TR, Parcela, Brazil). Samples were air-dried for approximately 48 h and cleaned. Grain moisture was measured using a moisture tester (AgraTronix MT-PRO, Streetsboro, OH, USA). Yield components included test weight (TW; kg hL^−1^), determined using a Hectoliter weight balance (Dalle mole, model 40, Caxias do Sul, Brazil) according to AACC Method 55-10.01 [58], and thousand kernel weight, measured by counting 100 seeds, weighing them with an electronic balance (Shimadzu model BL 3200H, Kioto, Japan) with an accuracy of 0.001 g, and multiplying the result by 10 to give a weight of 1000 kernels (TKW; expressed in g).

#### 4.5.4. Physicochemical and Rheological Quality of Wheat Samples

From each experimental plot, 40–80 g of grain were milled in a hammer mill (LabMill3100; Perten Instruments, Huddinge, Sweden) to obtain whole flour. Protein, ash content, falling number, gluten, and flour color were determined in whole wheat flour according to AACC Method 26-10.02 [58].

Grain protein content (PC), on a dry basis, was determined in the whole flour by NIR spectroscopy (XDS-Rapid Content Analyzer, FOSS NIR Systems, Hilleroed, Denmark), equipped with a dual-detection monochromator (model 6500, Hillerod, Denmark) monochromator with a measuring range: silicon 400–1100 nm and lead sulfide 1100–2500 nm, and ISIScan^TM^ software 1.2 (Infrasoft International LLC, State College, PA, USA), following AACC Method 39-10.01 [58]. Ash content (AC), on a dry basis, was obtained following ICC Method 104/1 [59], using a muffle furnace at 900 °C for 2.5 h.

The grain falling number (GFN) was determined using an FN 1900 (Perten Instruments, Stockholm, Sweden) according to AACC Method 56-81.03 [58], based on the liquefaction of starch gel by alpha-amylase. Gluten content was evaluated with a Glutomatic System (Perten, Stockholm, Sweden), following AACC Method 38-12.02 [58], reporting the parameter gluten index (GI).

Whole-flour color was analyzed using a colorimeter (CR-410 Chroma Meter, Minolta, Osaka, Japan), D_65_ illuminant, a Ø 50 mm measurement area, and a 10° viewing angle [60] [Konica Minolta, 2024]. Results were expressed according to the CIEL*a*b* system, using the following parameters: L*, lightness (0: dark, 100: white); chromaticity coordinates: a* (−60: green, +60: red) and b* (−60: blue, +60: yellow), C* (chroma or color intensity), and h* (hue or color tonality).

Rheological properties were obtained using a farinograph (50 g bowl Typ 820600 model, Brabender GmbH & Co. KG, Duisburg, Germany) according to AACC Method 54-21.02 [58], including water absorption (WA), dough development time (DDT), dough stability (DS), time to breakdown (TB), farinograph quality number (FQN), and mixture tolerance index (MTI). The data are provided as Supplementary Information (Figure S3).

### 4.6. Data Analysis

Data were analyzed separately by year using R version 4.0.4 (RStudio 2021). TKW, TW, maximum quantum yield of primary photochemistry (F_v_/F_m_), and wheat technological quality variables (AC, GFN, PC, GI, and whole wheat flour color) were analyzed using generalized linear models. Model assumptions were evaluated using residual-versus-fitted value plots to assess homoscedasticity, Q–Q plots to examine normality, and the ‘simulateResiduals’ function from the DHARMa package to further verify the normality of residuals. Estimated marginal means were obtained with the ‘emmeans’ package, and pairwise comparisons were performed with Tukey’s test using the ‘cld’ function. Correlation among AUDPCs, yield components, technological quality traits, and F_v_/F_m_ were computed using the ‘corrplot’ package.

## 5. Conclusions

During the wheat crop cycle, infection by leaf and spike diseases reduced yield components and directly affected the photosynthetic process. The maximum quantum yield of photosystem II (F_v_/F_m_) during the dough development stage (ZGS80) accurately reflected disease damage under field conditions. The interaction between the fungicide application and N rate mitigated physiological stress, preserved higher F_v_/F_m_ values and enhanced yield components (TW and TKW). This effect was particularly evident in the susceptible cultivar, which achieved the greatest yield gains when fungicide was combined with the recommended N rate, mainly due to reduced disease severity. The fungicide–nitrogen interaction also influenced grain quality, increasing protein and gluten contents while reducing ash content. However, the moderately resistant cultivar, despite superior field performance, produced lower grain protein content, that the susceptible cultivar. The effect of fungicide on grain protein content was most pronounced at low N rates, whereas at recommended or high N rates, its contribution was smaller. Nitrogen application was additionally associated with changes in flour color, reflecting increases in GPC, wheat flour AC, and grain falling number (GFN). Fungicide application improved flour appearance by enhancing lightness and yellowish tones. Overall, disease management using a moderately resistant cultivar sustained yield component, while the recommended N rate maintained adequate protein levels. For the susceptible cultivar, fungicide application was essential to mitigate disease impact and to maintain wheat technological quality.

## Figures and Tables

**Table 1:** Generalized linear model analyses of the fungicide (FU), cultivar (C), nitrogen rate (NR), and their interactions on thousand kernel weight (TKW), test weight (TW), ash content (AC), grain falling number (GFN), grain protein content (PC), gluten index (GI), and maximum efficiency of PSII photochemistry (F_v_/F_m_) during the 2019 growing season.

Growing Seasons	Source	df	Wheat Grain Technological Quality Parameters						Maximum Quantum Yield of Primary Photochemistry (F_v_/F_m_)
			TKW (g)	TW (kg hL^−1^)	AC	GFN	PC	GI	
2019	C								
	TBIO Audaz		32.0 ± 0.19 a	76.2 ± 0.53 a	1.58 ± 0.01 b	339 ± 2.91 b	14.5 ± 0.11 b	97.1 ± 1.84 a	0.68 + 0.03 a
	TBIO Tibagi		27.1 ± 0.19 b	73.1 ± 0.53 b	1.77 ± 0.01 a	230 ± 2.91 a	15.8 ± 0.11 a	68.4 ± 1.84 b	0.49 + 0.03 b
	FU								
	Sprayed (S)		32.0 ± 0.16 a	77.5 ± 0.53 a	1.61 ± 0.01 b	288 ± 2.91	15.1 ± 0.11	82.0 ± 1.84	0.62 + 0.03
	Not Sprayed (NFS)		27.0 ± 0.16 b	71.9 ± 0.53 b	1.74 ± 0.01 a	281 ± 2.91	15.2 ± 0.11	83.5 ± 1.84	0.55 + 0.03
	NR								
	Low N		28.7 ± 0.19 b	75.1 ± 0.45	1.70 ± 0.02	285 ± 3.56	14.2 ± 0.14 c	79.2 ± 2.25 a	0.56+ 0.04
	Recommended N		29.3 ± 0.19 b	74.5 ± 0.19 b	1.67 ± 0.02	284 ± 3.56	15.2 ± 0.14 b	80.2 ± 2.25 b	0.59 + 0.04
	High N		30.5 ± 0.19 a	74.4 ± 0.45	1.66 ± 0.02	285 ± 3.56	16.1 ± 0.14 a	89.0 ± 2.25 b	0.61 + 0.04
	*p* values								
	C	1	0.0001 **	0.0001 **	0.0001 **	0.0001 **	0.0001 **	0.0001 **	0.0001 **
	FU	1	0.0001 **	0.0001 **	0.0001 **	ns	ns	ns	ns
	NR	2	0.0001 **	0.030 *	ns	ns	0.0001 **	0.0001 **	ns
	C × FU	1	0.001 *	0.0001 **	0.025 *	ns	0.023 *	ns	ns
	C × NR	2	0.024 *	0.027 *	ns	ns	0.017 *	0.0001 **	0.03 *
	F × NR	2	0.03 *	ns	ns	ns	ns	ns	ns
	C × F × NR	2	ns	ns	ns	ns	ns	ns	ns

Means followed by the same letter within the same source of variation are not statistically different (Tukey’s test, *p* < 0.05). ** *p* ≤ 0.001 highly significant; * *p* ≤ 0.01 significant; ns *p* > 0.05 not significant.

**Table 2:** Generalized linear model analyses of the fungicide (FU), cultivar (C), nitrogen rate (NR), and their interactions on thousand kernel weight (TKW), test weight (TW), ash content (AC), grain falling number (GFN), grain protein content (PC), gluten index (GI), and maximum efficiency of PSII photochemistry (F_v_/F_m_) during the 2020 growing season.

Growing Seasons	Source	df	Wheat Grain Technological Quality Parameters						Maximum Quantum Yield of Primary Photochemistry (F_v_/F_m_)
			TKW (g)	TW (kg hL^−1^)	AC	GFN	PC	GI	
2020	C								
	TBIO Audaz		31.9 ± 0.18 a	76.8 ± 0.24 a	1.84 ± 0.01 b	423 ± 5.28 a	14.6 ± 0.06 b	99.2 ± 1.81 a	0.73 + 0.01 a
	TBIO Tibagi		24.2 ± 0.18 b	71.8 ± 0.24 b	1.92 ± 0.01 a	197 ± 5.28 b	16.8 ± 0.06 a	75.7 ± 1.81 b	0.66 + 0.01 b
	FU								
	Sprayed (S)		32.1 ± 0.18 a	76.4 ± 0.24 a	1.80 ± 0.01 b	312 ± 5.28 a	15.1 ± 0.06 b	83.5 ± 1.81 b	0.75 + 0.01 a
	Not Sprayed (NFS)		23.9 ± 0.18 b	72.0 ± 0.24 b	1.95 ± 0.01 a	308 ± 5.28 b	16.4 ± 0.06 a	91.4 ± 1.81 a	0.64 + 0.01 b
	NR								
	Low N		27.2 ± 0.22 b	73.5 ± 0.29	1.92 ± 0.01 a	315 ± 6.47	15.1 ± 0.07 c	83.7 ± 2.22	0.69 + 0.01
	Recommended N		28.1 ± 0.22 ab	74.1 ± 0.29	1.86 ± 0.01 ab	303 ± 6.47	15.9 ± 0.07 b	86.3 ± 2.22	0.70 + 0.01
	High N		28.2 ± 0.22 a	75.1 ± 0.29	1.84 ± 0.01 b	315 ± 6.47	16.2 ± 0.07 a	92.3 ± 2.22	0.70 + 0.01
	*p* values									
	C	1	0.0001 **	0.0001 **	0.0001 **	0.0001 **	0.0001 **	0.0001 **	0.0001 **
	FU	1	0.0001 **	0.0001 **	0.001 **	0.0001 **	0.0001 **	0.007 **	0.0001 **
	NR	2	0.03 *	ns	0.01 *	ns	0.0001 **	ns	ns
	C × FU	1	0.0001 **	0.0001 **	0.0001 **	0.036 *	0.0001 **	0.01 *	ns
	C × NR	2	0.01 *	0.023 *	ns	ns	ns	ns	ns
	F × NR	2	ns	ns	ns	ns	ns	ns	0.035 *
	C × F × NR	2	ns	ns	ns	ns	ns	ns	ns

Means followed by the same letter within the same source of variation are not statistically different (Tukey’s test, *p* < 0.05). ** *p* ≤ 0.001 highly significant; * *p* ≤ 0.01 significant; ns—not significant (*p* > 0.05).

**Table 3:** Generalized linear model analyses of the effects of fungicide (FU), cultivar (C), nitrogen rate (NR), and their interactions on whole wheat flour color parameters: lightness (L*), a* and b* chromaticity coordinates, chroma (C*), and hue (h*) during the 2019 growing season.

Growing Season	Source	df	Flour Color Parameters				
			L*	a*	b*	C*	h*
2019	C						
	TBIO Audaz		80.16 ± 0.07 b	3.46 ± 0.01 a	13.48 ± 0.03 a	13.92 ± 0.03 a	75.62 ± 0.07 b
	TBIO Tibagi		84.24 ± 0.07 a	2.61 ± 0.01 b	10.41 ± 0.03 b	10.74 ± 0.03 b	75.93 ± 0.07 a
	FU						
	Sprayed (S)		82.69 ± 0.07 a	2.86 ± 0.02 b	11.7 ± 0.03 b	12.1 ± 0.03 b	76.26 ± 0.07 a
	Not Sprayed (NFS)		81.69 ± 0.07 b	3.21 ± 0.02 a	12.2 ± 0.03 a	12.6 ± 0.03 a	75.29 ± 0.07 b
	NR						
	Low N		82.52 ± 0.11 a	2.96 ± 0.02 b	12.0 ± 0.03	12.4 ± 0.04	76.11 ± 0.09 a
	Recommended N		82.18 ± 0.11 a	3.04 ± 0.02 a	11.9 ± 0.03	12.4 ± 0.04	75.75 ± 0.09 b
	High N		81.96 ± 0.11 b	3.10 ± 0.02 a	11.9 ± 0.03	12.3 ± 0.04	75.46 ± 0.09 b
	*p* values						
	C	1	0.0001 **	0.0001 **	0.0001 **	0.0001 **	0.007 *
	FU	1	0.0001 **	0.0001 **	0.0001 **	0.0001 **	0.0001 **
	NR	2	0.0001 **	0.0001 **	ns	ns	0.0001 **
	C × FU	1	ns	ns	0.0001 **	0.0001 **	0.005 *
	C × NR	2	ns	ns	ns	ns	ns
	F × NR	2	ns	0.007 *	0.002 *	0.001 *	ns
	C × F × NR	2	ns	ns	ns	ns	ns

Means followed by the same letter within the same source of variation are not statistically different (Tukey’s test, *p* < 0.05). ** *p* ≤ 0.001 highly significant; * *p* ≤ 0.01 significant; ns—not significant (*p* > 0.05).

**Table 4:** Generalized linear model analyses of the effects of fungicide (FU), cultivar (C), nitrogen rate (NR), and their interactions on whole wheat flour color parameters: lightness (L*), a* and b* chromaticity coordinates, chroma (C*), and hue (h*) during the 2020 growing season.

Growing Season	Source	df	Flour Color Parameters				
			L*	a*	b*	C*	h*
2020	C						
	TBIO Audaz		80.0 ± 0.26 b	3.56 ± 0.06 a	12.5 ± 0.09 a	13.1 ± 0.10 a	74.3 ± 0.27 b
	TBIO Tibagi		82.7 ± 0.26 a	2.85 ± 0.06 b	10.7 ± 0.09 b	11.1 ± 0.10 b	75.1 ± 0.27 a
	FU						
	Sprayed (S)		82.1 ± 0.26 a	3.02 ± 0.06 b	11.3 ± 0.09 a	11.7 ± 0.10 b	75.2 ± 0.20 a
	Not Sprayed (NFS)		80.6 ± 0.26 b	3.40 ± 0.06 a	12.0 ± 0.09 b	12.5 ± 0.10 a	74.2 ± 0.20 b
	NR						
	Low N		81.1 ± 0.32	3.15 ± 0.07	11.8 ± 0.12	12.3 ± 0.13	74.6 ± 0.25
	Recommended N		81.4 ± 0.32	3.19 ± 0.07	11.6 ± 0.12	12.1 ± 0.13	74.7 ± 0.25
	High N		81.5 ± 0.32	3.27 ± 0.07	11.5 ± 0.12	11.9 ± 0.13	74.8 ± 0.25
	*p* values						
	C	1	0.0001 **	0.0001 **	0.0001 **	0.0001 **	0.001 *
	FU	1	0.0001 **	0.0001 **	0.0001 **	0.0001 **	0.001 *
	NR	2	ns	ns	ns	ns	ns
	C × FU	1	0.01 *	0.026 *	ns	ns	0.004 *
	C × NR	2	ns	ns	ns	ns	ns
	F × NR	2	ns	ns	ns	ns	ns
	C × F × NR	2	ns	ns	ns	ns	ns

Means followed by the same letter within the same source of variation are not statistically different (Tukey’s test, *p* < 0.05). ** *p* ≤ 0.001 highly significant; * *p* ≤ 0.01 significant; ns—not significant (*p* > 0.05).

## Data Availability

Data Availability Statements are available at https://osf.io/pv9s4/?view_only=b5f481b569734790babbdaf1328eb547, accessed on 1 December 2025.

## Supplementary Materials

The following supporting information can be downloaded from https://www.mdpi.com/article/10.3390/plants15050688/s1, Figure S1: F_v_/F_m_ assessment at different growth stages of wheat (TBIO Audaz) under fungicide application (A,C,E) and unsprayed conditions (B,D,F).; Figure S2: F_v_/F_m_ assessment at different growth stages of wheat (TBIO Tibagi) under fungicide application (A,C,E) and unsprayed conditions (B,D,F).; Figure S3: Rheological analysis considering farinograph parameters 2019 (A,C) and 2020 (B,D): water absorption (WA), dough development time (DDT), dough stability (DS), time to breakdown (TB), farinograph quality number (FQN), and mixture tolerance index (MTI) of TBIO Audaz and TBIO Tibagi with fungicide sprayed (S) and no fungicide sprayed (NFS) with three nitrogen levels (LN: 70 kg ha^−1^, RN: 130 kg ha^−1^, HN: 200 kg ha^−1^); Table S1: Soil characteristics of the experiments during 2019 and 2020; Table S2: Mean of temperature, humidity and total precipitation (July to November) during 2019 and 2020 growing seasons.
