# Supplementary material for: Disease Management Maintains Adequate Chlorophyll a Fluorescence and Enhances Wheat Grain Technological Quality
Source: Plants (Basel). 2026 Feb 25;15(5):688. doi: 10.3390/plants15050688 (PMC12987033; doi:10.3390/plants15050688)
Supplement: Supplementary file 1 [file plants-15-00688-s001.zip › plants-4114932-supplementary/plants-4114932-supplementary.pdf]

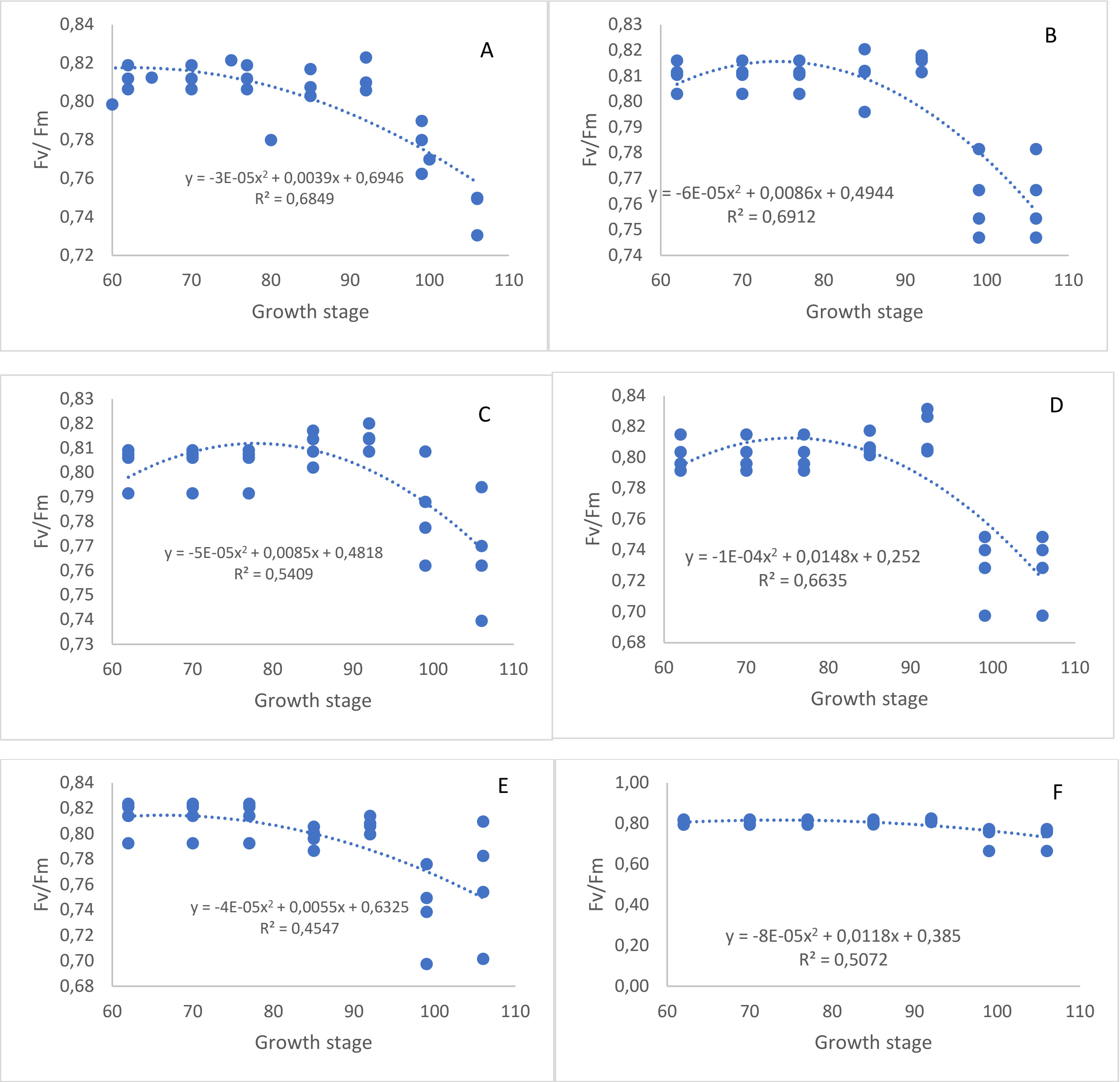

Figure S1. Fv/Fm assessment at different growth stages of wheat (TBIO Audaz) under fungicide application (A, C, E) and unsprayed conditions (B, D, F).

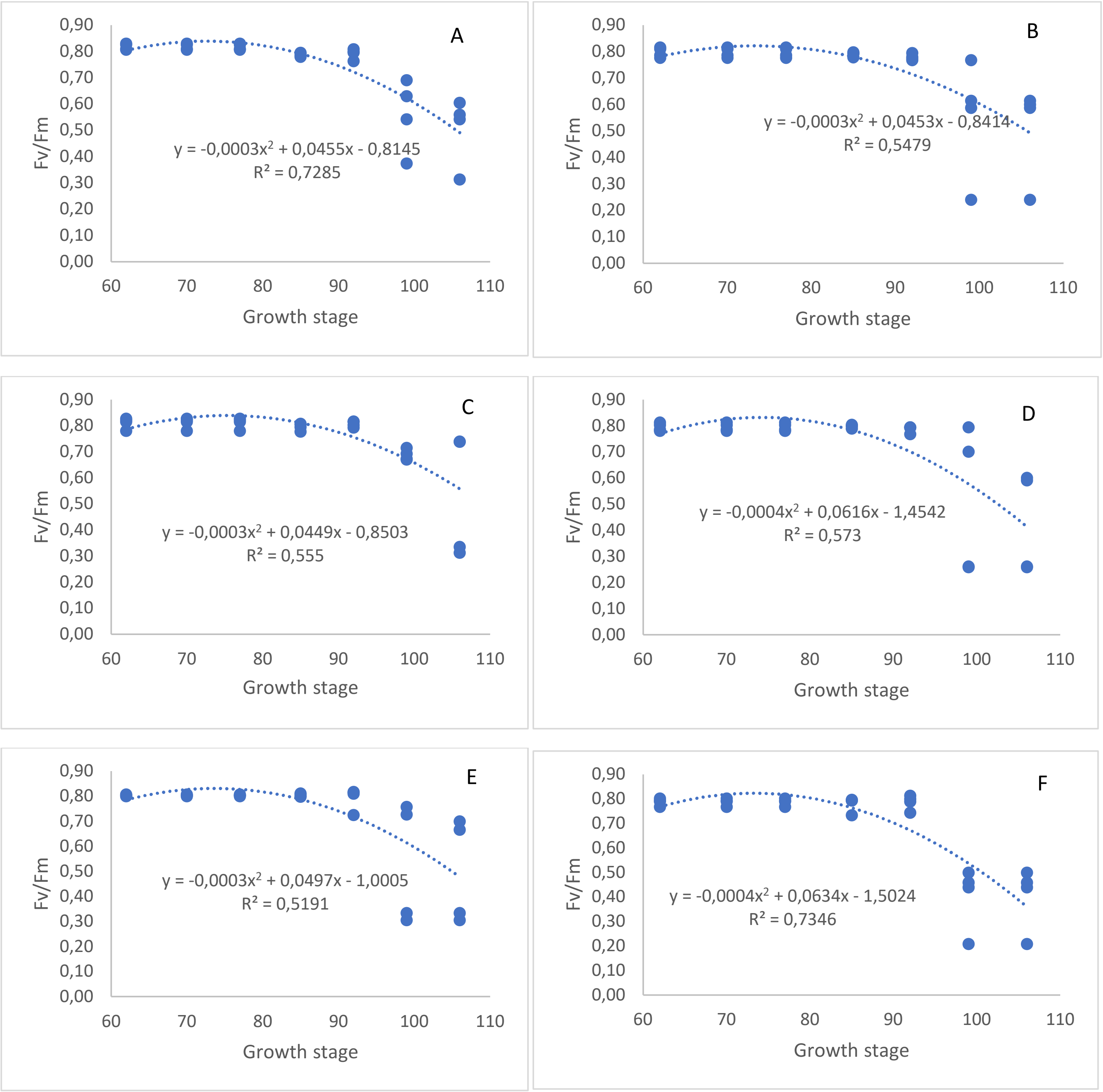

Figure S2. Fv/Fm assessment at different growth stages of wheat (TBIO Tibagi) under fungicide application (A, C, E) and unsprayed conditions (B, D, F).

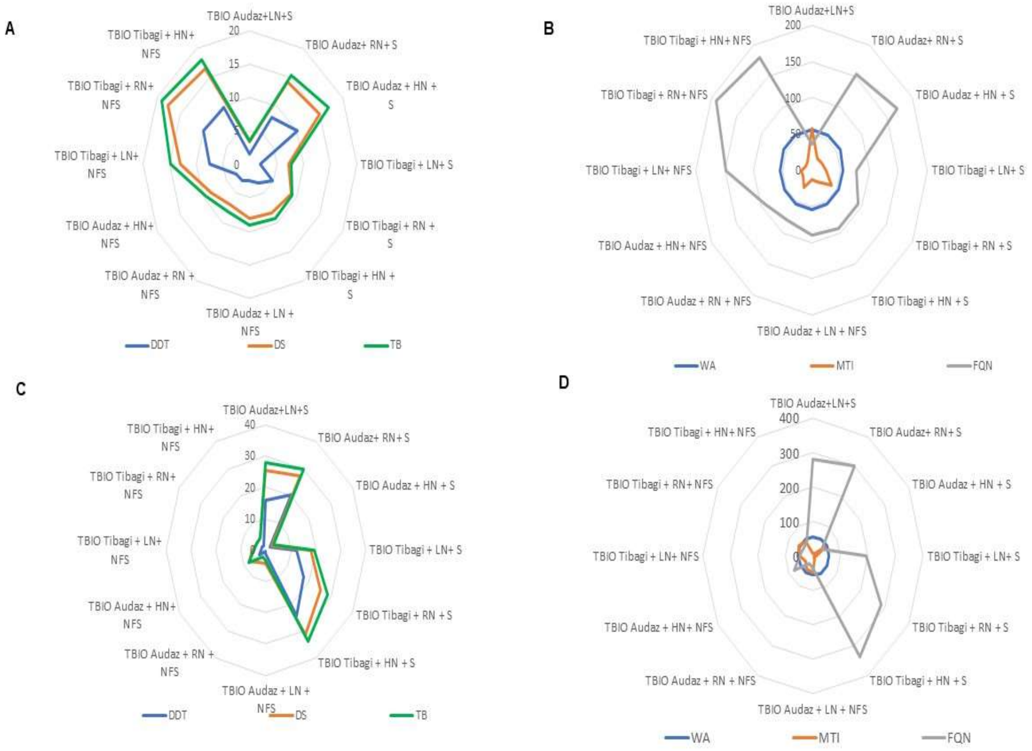

Figure S3 Rheological analysis considering farinograph parameters 2019 (A, C) and 2020 (B, D): water absorption (WA), dough development time (DDT), dough stability (DS), time to breakdown (TB), farinograph quality number (FQN), and mixture tolerance index (MTI) of TBIO Audaz and TBIO Tibagi with fungicide sprayed (S) and no fungicide sprayed (NFS) with three nitrogen levels (LN: 70 kg ha^{-1}, RN: 130 kg ha^{-1}, HN: 200 kg ha^{-1}).
