# Supplementary material for: Disease Management Maintains Adequate Chlorophyll a Fluorescence and Enhances Wheat Grain Technological Quality
Source: Plants (Basel). 2026 Feb 25;15(5):688. doi: 10.3390/plants15050688 (PMC12987033; doi:10.3390/plants15050688)
Supplement: Supplementary file 1 [file plants-15-00688-s001.zip › plants-4114932-supplementary/Table S1.docx]

**Table S1** Soil characteristics of the experiments during 2019 and 2020.

| **Soil characteristics** | **2019** | **2020** |
| --- | --- | --- |
| Organic matter (%) | 1 | 1,79 |
| Nitrogen (g kg^-1^) | 0,73 | 0,93 |
| NO_3_ (g kg^-1^) | 3,23 | 4,11 |
| NH_4_ (g kg^-1^) | 0,94 | 1,19 |
| Phosphorus (ppm) | 20 | 14 |
| Potassium (mg dm^-3^) | 99 | 115 |
| Calcium (cmolc.dm^-^³) | 1,7 | 1,8 |
| Magnesium (cmolc.dm^-^³) | 1,2 | 1,3 |
| Copper (mg dm^-3^) | 1 | 0,9 |
| Zinc (mg dm^-3^) | 1,1 | 0,8 |
| Manganese (mg dm^-3^) | 12,7 | 21 |
| pH H_2_O | 5,7 | 6 |

NO_3_: nitrate; NH_4_^+^: ammonium
