# Supplementary material for: Disease Management Maintains Adequate Chlorophyll a Fluorescence and Enhances Wheat Grain Technological Quality
Source: Plants (Basel). 2026 Feb 25;15(5):688. doi: 10.3390/plants15050688 (PMC12987033; doi:10.3390/plants15050688)
Supplement: Supplementary file 1 [file plants-15-00688-s001.zip › plants-4114932-supplementary/Table S2.docx]

**Table S2** Mean of temperature, humidity and total precipitation (July to November) during 2019 and 2020 growing seasons.

| **Month** | **2019** | | | | | **2020** | | | | |
| --- | --- | --- | --- | --- | --- | --- | --- | --- | --- | --- |
|  | **Temperature maximum** | **Temperature minimum** | **Temperature mean** | **Humidity** | **Rainfall** | **Temperature maximum** | **Temperature minimum** | **Temperature mean** | **Humidity** | **Rainfall** |
|  | **(ºC)** | **(ºC)** | **(ºC)** | **(%)** | **(mm)** | **(ºC)** | **(ºC)** | **(ºC)** | **(%)** | **(mm)** |
| July | 17.2 | 4.3 | 11.6 | 87.3 | 130.8 | 17.2 | 4.3 | 11.8 | 83.5 | 103.1 |
| August | 19.6 | 9.6 | 12.5 | 86.1 | 103 | 19.2 | 10.0 | 13.7 | 84.5 | 49.4 |
| September | 18.7 | 10.4 | 14.6 | 83 | 147.8 | 18.67 | 10.41 | 14.6 | 86.6 | 184.2 |
| October | 21.9 | 13.8 | 18.6 | 85.1 | 264.9 | 21.85 | 17.27 | 17.3 | 82.8 | 116.7 |
| November | 24.5 | 15.7 | 21.1 | 79.1 | 101.7 | 24.50 | 19.85 | 19.8 | 79.1 | 29.1 |
| **Mean** | 20.4 | 10.8 | 15.7 | 84.1 | 748.2* | 20.3 | 12.4 | 15.4 | 83.3 | 482.5* |

*****total
